# Expression profiling of immune inhibitory Siglecs and their ligands in patients with glioma

**DOI:** 10.1007/s00262-019-02332-w

**Published:** 2019-04-05

**Authors:** Kim C. M. Santegoets, Paul R. Gielen, Christian Büll, Barbara M. Schulte, Esther D. Kers-Rebel, Benno Küsters, Sandra A. J. F. H. Bossman, Mark ter Laan, Pieter Wesseling, Gosse J. Adema

**Affiliations:** 1 Radiotherapy and OncoImmunology Laboratory, Department of Radiation Oncology, Radboud Institute for Molecular Life Sciences, Radboud University Medical Center, Geert Grooteplein Zuid 32, 6525 GA Nijmegen, The Netherlands; 2 Department of Pathology, Radboud University Medical Center, Nijmegen, The Netherlands; 3 Department of Neurosurgery, Radboud University Medical Center, Nijmegen, The Netherlands; 4 Department of Pathology, VU University Medical Center, Amsterdam, The Netherlands; 5 Prinses Máxima Center for Pediatric Oncology and University Medical Center Utrecht, Utrecht, The Netherlands

**Keywords:** Myeloid-derived suppressor cells, Glioma, Siglecs, Sialic acids

## Abstract

Gliomas appear to be highly immunosuppressive tumors, with a strong myeloid component. This includes MDSCs, which are a heterogeneous, immature myeloid cell population expressing myeloid markers Siglec-3 (CD33) and CD11b and lacking markers of mature myeloid cells including MHC II. Siglec-3 is a member of the sialic acid-binding immunoglobulin-like lectin (Siglec) family and has been suggested to promote MDSC expansion and suppression. Siglecs form a recently defined family of receptors with potential immunoregulatory functions but only limited insight in their expression on immune regulatory cell subsets, prompting us to investigate Siglec expression on MDSCs. We determined the expression of different Siglec family members on monocytic-MDSCs (M-MDSCs) and polymorphnuclear-MDSCs (PMN-MDSCs) from blood of glioma patients and healthy donors, as well as from patient-derived tumor material. Furthermore, we investigated the presence of sialic acid ligands for these Siglecs on MDSCs and in the glioma tumor microenvironment. Both MDSC subsets express Siglec-3, -5, -7 and -9, with higher levels of Siglec-3, -7 and -9 on M-MDSCs and higher Siglec-5 levels on PMN-MDSCs. Similar Siglec expression profiles were found on MDSCs from healthy donors. Furthermore, the presence of Siglec-5 and -9 was also confirmed on PMN-MDSCs from glioma tissue. Interestingly, freshly isolated glioma cells predominantly expressed sialic acid ligands for Siglec-7 and -9, which was confirmed in situ. In conclusion, our data show a distinct Siglec expression profile for M- and PMN-MDSCs and propose possible sialic acid–Siglec interactions between glioma cells and MDSCs in the tumor microenvironment.

**Electronic supplementary material:**

The online version of this article (10.1007/s00262-019-02332-w) contains supplementary material, which is available to authorized users.

## Introduction

Gliomas are the most common primary brain tumors in adults arising from (precursors of) glial cells [2]. Most of these tumors show extensive (‘diffuse’) infiltration in the brain parenchyma, which precludes complete surgical resection. Even after state-of-the-art radio- and chemotherapy following surgical resection to the maximum feasible extent, patients with a glioblastoma (i.e., by far the most frequent and most malignant form of astrocytoma) have a dismal prognosis of only 14–16 months [3, 4].

At the moment, there are no effective FDA-approved treatment options available for radio- and chemotherapy-resistant gliomas. Cancer immunotherapy, where the patient’s own immune system is used to eradicate tumor cells, may provide a promising approach to specifically destroy glioma cells without harming the surrounding brain tissue. Numerous immunotherapies are currently being investigated in patients with glioma, but so far, the induction of tumor cell-specific cytotoxic T cells and survival benefit are only found in a minority of patients [5–7]. Gliomas often show a strong immunosuppressive tumor microenvironment with many suppressive immune cells including myeloid-derived suppressor cells (MDSCs), tumor-associated macrophages and regulatory T cells. This hampers immune cell function and limits the efficacy of cancer (immuno)therapies [7–10]. Therefore, combination therapies are now being actively investigated aiming to activate the immune system and to limit the immunosuppressive tumor microenvironment, in which myeloid cells are thought to play an important role.

We and others have previously reported an increase of MDSCs in the blood of glioma patients [11–13]. MDSCs express the myeloid lineage markers Siglec-3 (CD33) and CD11b but lack markers of mature myeloid cells (MHC II molecules HLA-DR,DP,DQ) [14, 15]. There are two main MDSC populations, a monocytic (M-) and a polymorphonuclear (PMN-) MDSC population, which are defined by the expression of CD14 or CD15, respectively. In addition, a third subset has been suggested that is lacking the myeloid cell lineage markers CD15 and CD14. This is a mixed population of early progenitor cells and is, therefore, called early-stage MDSCs (e-MDSCs) [14, 16].

Both M-MDSCs and PMN-MDSCs are elevated in the blood of glioma patients, with PMN-MDSCs being the main MDSC population detected in the tumor tissue [11, 17]. We have previously shown that PMN-MDSCs from glioma patients are potent inhibitors of T-cell function that possibly contributes to the immunosuppressive microenvironment [13]. To interfere with their immunosuppressive function, more knowledge about the induction of MDSCs and their suppressive phenotype is needed. One of the cell surface markers used to identify MDSCs is Siglec-3, a member of the sialic acid-binding immunoglobulin-like lectin (Siglec) family [18]. Interestingly, triggering of Siglec-3 in MDSCs has been shown to promote their expansion and induce the secretion of immunosuppressive cytokines [19].

The human Siglec family consists of 14 members and can be divided into two groups: the conserved Siglecs and the CD33-related Siglecs. Conserved Siglecs (-1, -2, -4, -15) show a high sequence similarity across species, whereas CD33-related Siglecs (-3, -5 to -11, -14 and -16) display high sequence variability across species [20–22]. Siglecs are broadly expressed throughout the immune system, but which Siglecs are expressed by MDSCs apart from Siglec-3 is largely unknown. Siglecs are type-I transmembrane proteins with an extracellular sialic acid-binding domain and most of them contain a cytoplasmic immunoreceptor tyrosine-based inhibitory motif (ITIM). Their ligands are sialic acid sugars which are located on glycans of membrane glycoproteins and glycolipids of virtually all cells in the human body. Sialic acids play an important physiological role at the molecular (e.g., regulation of glycoprotein stability and function) and cellular level (e.g., cell–cell interactions) [23, 24]. Sialic acids can interact with Siglecs on the same cell surface in *cis* or on other cells in *trans*. In general, the interaction between a Siglec with its sialic acid ligand initiates an ITIM-mediated suppressive signal [21, 22]. Thereby, Siglecs can dampen immune cell activation and possibly contribute to immunological homeostasis [21, 25]. Recent examples demonstrate the strong immunosuppressive capacity of Siglecs. For instance, sialic acid binding to Siglec-2 on B cells has been shown to suppress B-cell activation and induces tolerance to membrane antigens [26, 27]. Furthermore, we have recently shown that monocytic cell activation can be dampened via Siglec-3 triggering [28].

A growing body of evidence suggests that tumor cells exploit the Siglec family to modulate immune cell function and to evade detection by the immune system [29–36]. Tumor sialic acids have been shown to dampen NK cell-mediated killing via Siglec-7 and -9 as well as effector T-cell function [31, 32, 37]. Furthermore, triggering of Siglec-9 on macrophages by tumor-derived sialic acids induced a tumor-associated macrophage-like phenotype promoting immune suppression in the tumor microenvironment [33]. These studies suggest that tumor-derived sialic acids modulate the function of effector and regulatory immune cell subsets by interacting with the Siglec receptor family. To what extent sialic acid–Siglec interactions could influence MDSC function in the tumor microenvironment is largely unknown. Therefore, we assessed the expression of Siglec family members on MDSCs from glioma patients and healthy donors. In addition, the expression of putative *cis* and *trans* sialic acid ligands on MDSCs and glioma cells in the tumor microenvironment was analyzed.

We found that M- and PMN-MDSCs from blood of glioma patients and healthy donors express multiple Siglecs with specific expression profiles, while e-MDSCs show only little to no Siglec expression. This could be confirmed on glioma-infiltrating PMN-MDSCs. Subsequently, we identified expression of putative *trans* Siglec ligands on glioblastoma cell lines as well as in patient-derived glioma tissue. These data support possible sialic acid–Siglec interactions between MDSCs and glioma cells, which could contribute to the immunosuppressive role of MDSCs in glioma patients.

## Materials and methods

### Blood and tumor samples

Peripheral blood samples were collected from 14 healthy individuals and from 18 glioma patients undergoing neurosurgical resection or biopsy for intracranial tumors at the Radboud University Medical Center (Radboudumc). The mean age of the patients was 58 years (range 43–76 years) and 56% were males. The healthy donors were anonymous and not age and sex matched. All patients had histologically proven brain tumors diagnosed by neuropathologists of the Radboudumc. The tumors were classified according to the WHO 2016 Classification of tumors of the Central Nervous System [38], and encompassed 1 low-grade diffuse astrocytoma, Isocitrate dehydrogenase (IDH)-mutant (WHO grade II), 6 oligodendrogliomas, IDH-mutant and 1p/19q-codeleted (2 WHO grade II, 4 WHO grade III), 2 glioblastomas, IDH-mutant, and 9 glioblastomas, IDH-wildtype. Fresh tumor tissue samples were obtained from 6 patients by sonic aspiration [3 oligodendrogliomas (2 WHO grade II, 1 WHO grade III) and 3 glioblastomas (2 IDH-wildtype, 1 IDH-mutant)]. In addition, histological sections of formalin-fixed paraffin-embedded glioma tissue were obtained from 4 patients with glioblastoma (3 IDH-wildtype, 1 IDH-mutant). Ten patients had started a 3 × 5 mg dexamethasone regime on the day before surgery, while 8 patients received dexamethasone at an earlier time point to reduce edema. Immediately after the blood and fresh tumor tissue samples were obtained, processing of these samples was started. Flow cytometry measurements were performed within 24 h.

### Tissue handling

PBMCs were isolated from heparinized venous blood of patients and healthy donors using a lymphoprep gradient (Lucron Bioproducts, Gennep, the Netherlands). Fresh glioma material was obtained by ultrasonic aspiration or excision. Ultrasonic aspirates were collected in a sterile suction trap and tumor cell suspensions were prepared as described previously [11, 39]. Briefly, tumor fragments were washed extensively to discard blood and suction fluid. Then, tumor fragments were incubated with collagenase type-IA (50 µg/ml) (Sigma-Aldrich, St Louis, MO), DNAse type-I (10 μg/ml) (Roche, Mannheim, Germany) and trypsin inhibitor (1 μg/ml) (Sigma-Aldrich) in Hank’s Balanced Salt Solution (HBSS) (Gibco, Invitrogen, Leek, the Netherlands) at 37 °C followed by mechanical disruption by pipetting. To remove granulocytes, including mature neutrophils, tumor cell suspensions were placed on a lymphoprep gradient as was also used for PBMC isolation.

### Flow cytometry and sorting

Using standardized flow cytometry protocols as described previously, cells were stained for different membrane markers using regular antibodies as well as with lectins and recombinant Siglec-Fc chimeras to determine their sialic acid composition [11, 28]. To exclude dead cells, the cells were stained with fixable viability dye eFluor 780 (eBioscience, Vienna, Austria). Prior to staining, Fc receptors were blocked by incubation with 2% human serum. Membrane markers and Siglecs were stained with directly conjugated monoclonal antibodies (mAbs) for 20 min on ice. Fluorescent mAbs were obtained from BD (San Jose, CA) (CD33-APC, HLA-DR,DP,DQ-FITC, CD14-PerCP), Pelicluster (Amsterdam, the Netherlands) (CD14-PE), IO-Test (CD11b-PE), Biolegend (San Diego, CA) (CD15-PE-Cy7, CD45-PerCP, Siglec-5-PE, Siglec-7-PE, Siglec-10-PE), Sigma-Aldrich (Siglec-2-PE) and R&D Systems (Siglec-9-PE). Isotype controls were obtained from BD (IgG1-APC, IgG1-PE-Cy7, IgG2a-PE, IgG2a-FITC, and IgG1-PE) and Biolegend (IgG2a-PerCP). Cells were washed and resuspended in PBS with 1% BSA and 0.02% sodium azide and measured on a CyAn (Beckman Coulter, Brea, CA), FACSVerse (BD) or Gallios (Beckman Coulter) flow cytometer. For lectin staining, cells were stained with biotinylated lectins *Maackia amurensis* lectin II (MALII), *Sambucus nigra *agglutinin (SNA-I) and peanut agglutinin (PNA) in carbo-free blocking solution containing 1 mM CaCl^2+^ and 1 mM MgCl^2+^ (Vector Laboratories, Burlington, NO) for 40 min. After washing, the cells were incubated with PE-conjugated Streptavidin (BD, Franklin Lakes, NJ). For Siglec ligand staining, 0.4 µg/ml recombinant human Siglec-Fc chimera (R&D Systems, Minneapolis, MN) was pre-complexed with with Alexa Fluor 647-conjugated goat anti-human IgG (H + L) antibody (Life Technologies, Burlington, Ontario, Canada) in carbo-free blocking solution containing 1 mM CaCl^2+^ and 1 mM MgCl^2+^ and added to the cells for 40 min at 4 °C. Cells incubated with Alexa Fluor 647 goat anti-human IgG antibody only served as control. Data were analyzed using FlowJo 9.2 (Tree Star Inc, Ashland, OR) and events were gated on single viable cells for further analysis. Median fluorescence intensity (MFI) values of Siglec stainings were corrected based on the MFI values of the isotype controls to control for flow cytometer type. For sorting of MDSC subsets, PBMCs were stained with HLA-DR,DP,DQ-FITC, CD33-APC, CD14-PE and CD15-PE-Cy7 and sorted on a FACS Aria (BD). Data were analyzed using GraphPad Prism 5 software.

### Quantitative PCR

FACS-sorted cells were resuspended in RNA lysis buffer (Zymo Research, Irvine, CA) and stored at − 80 °C until RNA isolations were performed using the ZR RNA isolation kit (Zymo Research), according to the manufacturer’s instructions. The RNA was treated with DNAase I (amplification grade; Invitrogen) to remove any genomic DNA before being reverse-transcribed into cDNA, as described elsewhere [40]. To check for genomic DNA contamination control samples without reverse transcriptase were included. cDNA was stored at − 20 °C until further use. Real-time PCR was performed on a CFX96 system (Bio-Rad, Veenendaal, Netherlands) using SYBR Green reaction mix (Sigma-Aldrich) and Siglec expression was calculated relative to GAPDH expression. Primer sequences (Sigma-Aldrich) were derived from the Harvard Primer Bank database (Supplementary Table 1) [41].

### Glioma cell culture

Human glioma cell lines U-87 (HTB-14), T98G (CRL-1690) and U-251 were grown in Dulbecco’s Modified Eagle’s Medium (DMEM) with  GlutaMAX (Gibco) supplemented with 10% heat-inactivated FBS (Greiner Bio-one, Frickenhausen, Germany), and antibiotic–antimycotic solution (Life Technologies) to confluency and passaged using trypsin. The cells were used within 3 months after resuscitation and regularly screened for mycoplasma contamination using a detection kit (Lonza, Walkersville, MD). All cells were incubated in a humidified CO2 incubator at 37 °C.

### Immunohistochemistry

Siglec ligand expression on paraffin-embedded glioma tissue samples (4 μm) was assessed using human Siglec-Fc chimeras. Sections were deparaffinized and rehydrated with Xylene, graded ethanol and water. Sections were heated at 96 °C for 30 min in target retrieval solution, citrate pH 6.0 (Dako, Agilent Technologies, Santa Clara, CA). After cooling down to room temperature, sections were treated with 3% H_2_O_2_ in PBS for 15 min, washed with PBS and blocked with 20% goat serum. Siglec-7 and -9 ligands were stained with 50 nM Siglec-Fc chimeras, pre-complexed with 20 nM horseradish peroxidase-conjugated anti-human Fc antibody (Thermo Scientific, Waltham, MA) in HBSS at 4 °C. Human IgG1 (Sigma-Aldrich) was used as isotype control. Siglec-Fc binding was detected with a DAB peroxidase substrate kit (Vector Laboratories). All tissue sections were counterstained with hematoxylin, washed with water, dehydrated and mounted with KP-mounting medium (Klinipath, Olen, Belgium). Alternatively, sections were treated with 250 mU/ml *Clostridium perfringens* sialidase (Sigma-Aldrich) in HBSS for 2.5 h at 37 °C and washed with PBS before staining with Siglec-Fc chimeras. Images were acquired using a Leica DM6000 system (Leica, Wetzlar, Germany).

## Results

### Blood MDSCs express several Siglec family members

**Fig. 1:**
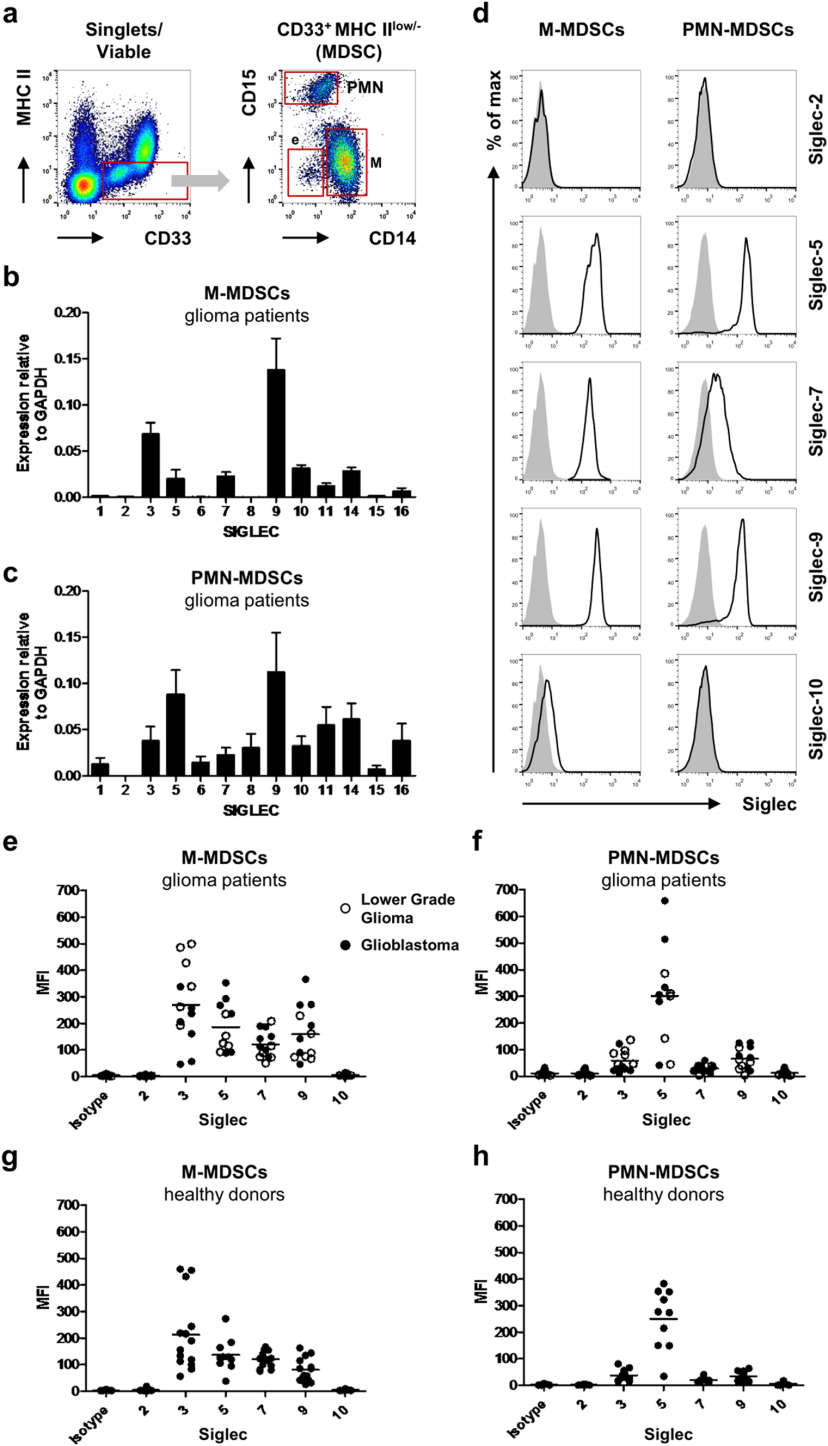
Siglec expression on blood MDSCs from glioma patients and healthy donors. **a** Gating strategy for MDSCs from blood, showing M-, PMN- and e-MDSC subsets. **b**, **c** qPCR analysis of Siglec expression in M-MDSCs (**b**) and PMN-MDSCs (**c**) from glioma patients. Bar diagrams show mean relative Siglec expression normalized to GAPDH ± SEM (*n* = 5). **d**–**h** Cell surface expression of Siglecs on MDSC subsets measured by flow cytometry. Representative histograms show cell surface Siglec expression on M-MDSCs (left panel) and PMN-MDSCs (right panel) (**d**). The gray histograms represent the isotype control. Dot plots show median fluorescence intensity (MFI) of cell surface Siglec expression of M-MDSCs and PMN-MDSCs derived from blood of glioma patients (*n* = 11–13; 6 lower-grade gliomas (open circles) and 7 glioblastomas (filled circles)) (**e**, **f**) as well as from healthy donors (*n* = 10–14) (**g**, **h**). Horizontal lines show the mean Siglec expression

Most myeloid cells express several members of the Siglec family, but not much is known about Siglec expression on MDSCs, except for Siglec-3. Therefore, we assessed the expression of the different Siglec family members on sorted M-MDSCs (CD33^+^ MHC II^low/−^ CD14^+^) and PMN-MDSCs (CD33^+^ MHC II^low/−^ CD15^+^) from the blood of glioma patients (Fig. 1a). In our patient cohort, M-MDSCs and PMN-MDSCs represented an average of 5.07% (SD = 6.27) and 3.28% (SD = 4.12) of PBMCs, respectively, which is in line with our previously reported data [11, 13]. RNA analysis confirmed Siglec-3 expression by both MDSC subsets. Additionally, M-MDSCs expressed mRNA for Siglec-5, -7, -9, -10, -11, -14, and -16 with Siglec-9 showing the highest expression (Fig. 1b). PMN-MDSCs showed a similar expression profile, but in contrast to M-MDSCs, they also expressed Siglec-6, and -8 at the mRNA level (Fig. 1c). Interestingly, expression of Siglec-5 was about 4 times higher in PMN-MDSCs compared to M-MDSCs. Next, we determined Siglec expression on the cell surface of M- and PMN-MDSCs from patients with glioblastomas and lower-grade gliomas by flow cytometry using available Siglec antibodies. As expected, Siglec-3 (CD33) was detected on the cell surface of both subpopulations, with a higher expression on M-MDSCs (Fig. 1a, e–f). Siglec-2, a conserved Siglec known to be B-cell specific, was included as a negative control and was not found on either M- or PMN-MDSCs (Fig. 1d–f) [21]. Siglec-5, -7 and -9 were clearly detected on both M- and PMN-MDSCs, with Siglec-5 being the most highly expressed on PMN-MDSCs (Fig. 1d–f). In contrast to the qPCR data, there was little to no expression of Siglec-10 detectable. Additional data have also shown a lack of Siglec-6 and -8 expression on both MDSC subsets (data not shown). These data confirm that both MDSC subsets express Siglec-3, -5, -7 and -9, with a generally higher Siglec expression on M-MDSCs compared to PMN-MDSCs, except for Siglec-5 which is highly expressed on PMN-MDSCs.

Healthy donors generally have very low amounts of MDSCs in their peripheral blood [11, 13, 16]. Nevertheless, we also determined Siglec expression on MDSCs present in healthy donors. M-MDSCs (0.53% (SD = 0.28) of PBMCs) and PMN-MDSCs (2.38% (SD = 2.61) of PBMCs) from healthy donors express the same Siglecs as detected on MDSC subsets from glioma patients, with similar expression profiles (Fig. 1g, h). Thus, both M-MDSCs and PMN-MDSCs express Siglec-3, -5, -7 and -9 in glioma patients as well as healthy donors, with a specific expression profile dependent on the MDSC subtype.

In addition to these M- and PMN-MDSC populations, we also analyzed Siglec expression on the CD15-CD14- e-MDSCs (Fig. 1a). In our cohort, the mean percentage of this population was 0.73% (SD = 0.50) in glioma patients and 2.12% (SD = 2.05) in healthy donors. The expression of Siglec-3 on these cells is similar to PMN-MDSCs. Other than that, they show little to no Siglec expression (Supplementary Fig. 1). Therefore, we focused on M- and PMN-MDSCs for further analyses.

### Glioma-infiltrating PMN-MDSCs express Siglec-5, -7, and -9

**Fig. 2:**
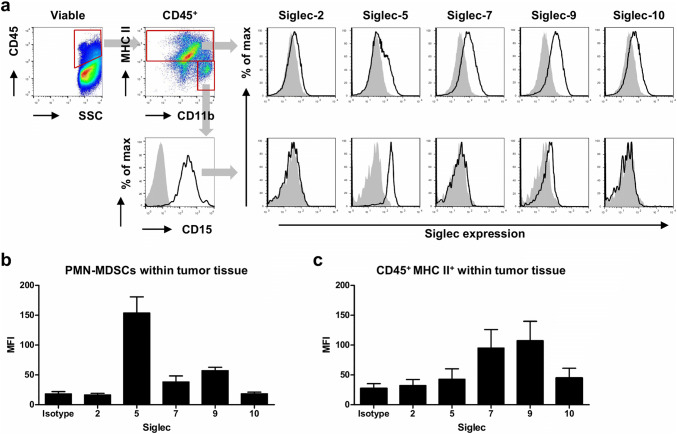
Siglec expression on glioma-infiltrating PMN-MDSCs. **a**–**c** Expression of Siglecs on the surface of PMN-MDSCs and myeloid cells in glioma tissue. Gating strategy shows CD45^+^ MHC II^+^ CD11b^+^ myeloid cells and CD45^+^ MHC II^−^ CD11b^+^ CD15^+^ PMN-MDSCs (**a**, left panels). Representative histograms show Siglec expression on myeloid cells (**a**, upper panel) and PMN-MDSCs (**a**, lower panel). The gray histograms represent the isotype control. **b**, **c** Bar diagrams show mean Siglec expressio*n* ± SEM on glioma-infiltrating PMN-MDSCs (**b**) and myeloid cells (**c**) from three glioma patients

We have previously characterized MDSC infiltration in glioma tissue and found mainly PMN-MDSCs [11, 13]. Therefore, we additionally analyzed Siglec expression on glioma-infiltrating PMN-MDSCs. Single cell suspensions derived from tumor tissue were stained for PMN-MDSC markers and Siglecs. Cells were first gated on single, viable cells and CD45 expression (Fig. 2a, left panel). Then, PMN-MDSCs were identified as CD11b^+^ MHC II^low/−^ CD15^+^, because no clear Siglec-3 signal above background could be detected on these tumor-infiltrating MDSCs, as described previously [11]. Similar to blood PMN-MDSCs, Siglec-5 and -9 could be detected on glioma-infiltrating PMN-MDSCs, with the highest expression of Siglec-5 (Fig. 2a, b). Low Siglec-7 expression could be detected on glioma-infiltrating PMN-MDSCs in some patients, while on others it was undetectable (Fig. 2a, b). Besides PMN-MDSCs, a group of CD45^+^ CD11b^+^ MHC II^+^ myeloid cells is present in glioma tissue that consists of tumor-infiltrating monocytes, tumor-associated macrophages and/or microglia. This group expressed Siglec-7 and -9 to a similar extent as CD33^+^ MCH II^+^ cells from blood, but displayed lower levels of Siglec-5 (Fig. 2a, c, Supplementary Fig. 2). Altogether, these data show that glioma-infiltrating PMN-MDSCs consistently express Siglec-5 and -9, while other myeloid cells in the tumor mainly express Siglec-7 and -9.

### MDSCs express *cis* Siglec ligands

**Fig. 3:**
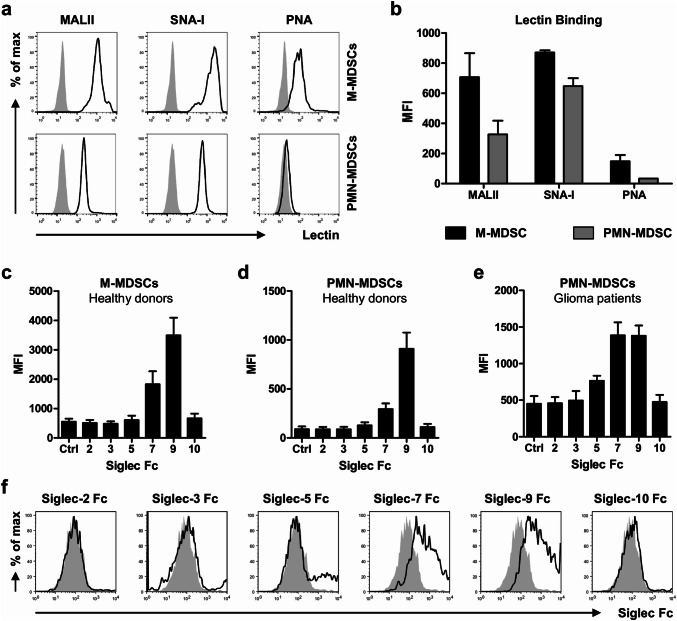
Expression of sialic acids on blood MDSCs from healthy donors and glioma patients. **a**, **b** Binding of sialic acid-binding lectins MALII (α2,3-linked sialic acid) and SNA-I (α2,6-linked sialic acid) and galactose-binding lectin PNA to MDSCs isolated from blood of healthy donors. **a** Representative histograms show lectin binding to M-MDSCs (upper panel) and PMN-MDSCs (lower panel) as determined by flow cytometry. The gray histograms represent the unstained control **b**) Bar diagram shows MFI ± SEM of lectin binding to both MDSC subsets (*n* = 3). **c**, **d** Binding of recombinant Siglec Fc chimera to MDSCs from healthy donors (*n* = 3). Bar diagrams shows MFI ± SEM of Siglec Fc binding to M-MDSCs (**c**) and PMN-MDSCs (**d**). **e**, **f** Siglec ligand expression on PMN-MDSCs obtained from the blood of glioma patients (*n* = 5). Bar diagram shows Siglec Fc binding as MFI ± SEM (**e**) and histograms show representative Siglec Fc binding (**f**). The gray histograms represent the isotype control

We next investigated the expression of sialic acids on MDSCs using the lectins MALII, SNA-I and PNA. These lectins preferentially recognize α2,3-linked or α2,6-linked sialic acids or glycans without sialic acids, respectively. M-MDSCs and PMN-MDSCs were gated as shown in Fig. 1a and analyzed for lectin binding by flow cytometry. Both MDSC subsets expressed α2,3-linked and α2,6-linked sialic acids, with the highest levels on M-MDSCs (Fig. 3a, b). Interestingly, also PNA binding to M-MDSCs, but not to PMN-MDSCs could be observed, indicating the presence of uncapped glycans on these cells. Next, we investigated which Siglecs could potentially form *cis* interactions with sialic acids present on the MDSCs. PBMCs from healthy donors were stained with recombinant human Siglec-Fc chimeras together with MDSC markers and analyzed by flow cytometry. From all Siglec-Fc chimeras tested, only Siglec-7 and Siglec-9 showed binding to both MDCS subsets, with Siglec-9 binding being the highest (Fig. 3c, d). In line with the plant-derived lectin stainings, M-MDSCs showed generally higher binding of Siglec-7 and -9 chimeras compared to PMN-MDSCs. Similar to the healthy donors, PMN-MDSCs from blood of glioma patients also expressed ligands for Siglec-7 and Siglec-9 (Fig. 3e, f). Here, the higher expression of Siglec-9 ligands compared to Siglec-7 ligands was not detected. Siglec-5 ligands were detected on PMN-MDSCs in 3 out of 5 glioma patients. No signal for Siglec-2, -3 or -10 binding was found. Altogether, our data show that both MDSC subsets express ligands for Siglec-7 and Siglec-9 that may form *cis* interactions with the corresponding Siglec receptors present on these cells.

### Glioblastoma cell lines and patient-derived glioma cells express trans Siglec ligands

**Fig. 4:**
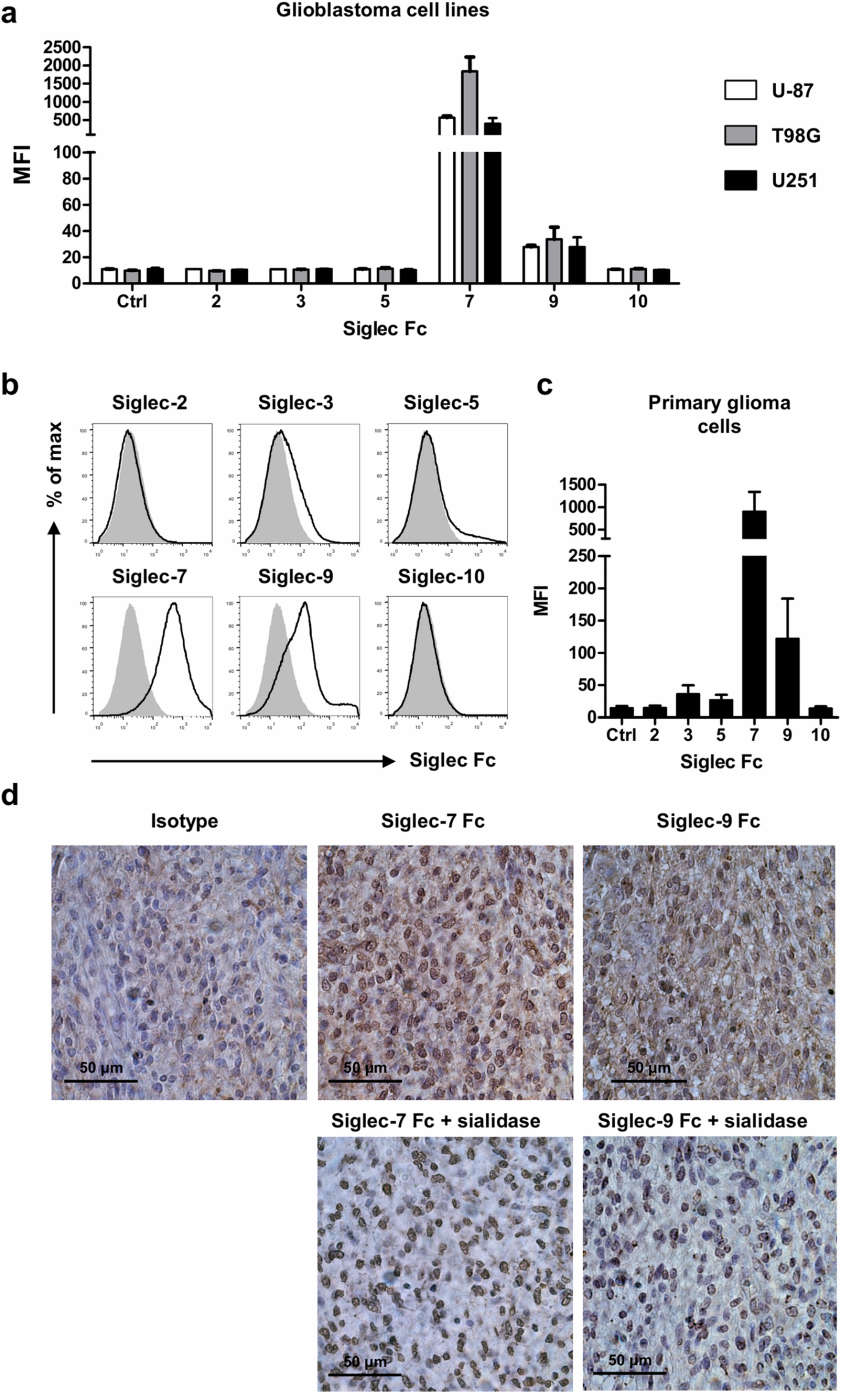
Glioma cell lines and freshly resected glioma cells express *trans* ligands for Siglecs. **a** Three glioblastoma cell lines were stained with recombinant Siglec Fc chimera and analyzed by flow cytometry. Bar diagrams show mean MFI ± SEM of Siglec Fc chimera binding to U-87, T98G and U251 cells of three independent experiments. **b**, **c** Expression of Siglec ligands on glioma cells obtained from freshly resected tumor tissue (glioblastoma *n* = 1, oligodendroglioma *n* = 3). Representative histograms show binding of Siglec Fc chimera to CD45^−^ cells isolated from glioma tissue (**b**). Bar graphs show mean fluorescent intensity ± SEM of Siglec Fc binding (**c**). **d** Immunohistochemistry for Siglec-7 and Siglec-9 ligand expression in paraffin-embedded glioma tissue untreated (upper row) or treated with sialidase (lower row). Data are representative of 4 glioblastoma patients

Many tumors show aberrantly high expression of sialic acid-carrying glycans that possibly interact with Siglecs expressed on MDSCs and other infiltrating immune cells. To investigate whether glioma cells express sialic acids that can serve as ligands for Siglecs, three different human glioblastoma cell lines (U-87, T98G and U251) were probed with recombinant human Siglec-Fc chimeras and analyzed by flow cytometry. While no staining of ligands for Siglec-2, -3, -5 and -10 was observed, ligands for Siglec-7 and -9 were detected on all three cell lines (Fig. 4a). Expression of Siglecs themselves on the cell surface of the glioma cell lines was not detected (data not shown).

Next, we investigated whether the results from glioblastoma cell lines resemble the Siglec ligand expression as it occurs on glioma cells in situ. Therefore, single cell suspensions were prepared from freshly isolated glioma tissue (from glioblastoma *n* = 1 and oligodendrogliomas *n* = 3) and stained with anti-CD45 antibody and recombinant human Siglec-Fc chimeras. Glioma cells were gated as single, viable cells lacking CD45 expression, the marker for infiltrating immune cells. On primary glioma cells, we mainly detected ligands for Siglec-7 and -9, while some patients also showed low levels of Siglec-3 and -5 ligand staining (Fig. 4b, c). The presence of the two most dominant ligands, for Siglec-7 and -9, in glioma tissue was confirmed by immunohistochemistry (Fig. 4d). Specificity of Siglec-7 and -9 Fc chimera binding was confirmed by the loss of signal after hydrolysis of sialic acids by sialidase. Staining did not completely disappear, probably due to aspecific staining caused by the sialidase [42].

These data show that mainly sialic acid ligands for Siglec-7 and -9 are expressed by glioblastoma cell lines and on patient-derived glioma tissue. Interestingly, Siglec-7 and -9 were present on both MDSC subsets found in the blood and expression of Siglec-9 was also confirmed on PMN-MDSCs from glioma tissue, suggesting possible *trans* interactions between glioma cells and MDSCs. In addition to MDSCs, other myeloid (regulatory) cells present in the tumor could interact with glioma cells via Siglec-7 and -9.

## Discussion

The presence of MDSCs is increased in the blood of patients with glioma in a grade-dependent manner and they infiltrate the glioma tissue [11, 13]. Siglec-3 is a widely used surface marker for MDSCs and has been suggested to promote MDSC expansion as well as their suppressive phenotype [18, 19, 21]. Expression of Siglec-3 on MDSCs was already well established, but expression of other Siglec family members on MDSCs has not been investigated so far. Here, we show that M-MDSCs and PMN-MDSCs isolated from the blood of glioma patients and healthy donors also express Siglec-5, -7 and -9 on their cell surface. M-MDSCs expressed higher Siglec-3, -7 and -9 levels compared to PMN-MDSCs, whereas Siglec-5 was highest on PMN-MDSCs. Expression of Siglec-5 and -9 was also confirmed on PMN-MDSCs derived from glioma tissue. E-MDSCs have only low Siglec expression. Additionally, we showed that glioma cells mainly express ligands for Siglec-7 and -9. These data imply that sialic acid–Siglec interactions between glioma cells and PMN-MDSCs in the tumor microenvironment can occur that could influence MDSC function.

For Siglec-3, -5, -7 and -9, we found mRNA transcripts as well as cell surface protein expression on both MDSC subsets. In addition, expression of Siglec-11, -14 and -16 was found on the mRNA level, but their expression on the cell surface is not yet confirmed. Siglec-10 expression was detected by qPCR in both M-MDSC and PMN-MDSCs, but protein expression could not be confirmed with the existing monoclonal antibodies. Also Siglec-6 and -8 membrane expression could not be detected. In general, M-MDSCs expressed higher levels of Siglecs compared to PMN-MDSCs, except for higher levels of Siglec-5 on PMN-MDSCs. Noteworthy, the antibody against Siglec-5 is cross-reactive to Siglec-14, but the higher expression of Siglec-5 in PMN-MDSCs was also found on mRNA level. The biological relevance of the different expression patterns of Siglec-3, -5, -7 and -9 between both MDSC subsets remains to be determined. In addition to the M- and PMN-MDSCs, we have also determined Siglec expression on the CD15-CD14- e-MDSCs. This population, however, is more prone to contamination by other cells, and therefore inclusion of the lineage markers CD3, CD19 and CD56 is important to characterize them as true lineage-negative MDSCs [14]. These lineage markers were not present in our MDSC panel and therefore, the levels of e-MDSCs found in our patients and controls might be an overestimation. We were able to include these lineage markers in four of our patients and could confirm low Siglec-3, -5 and -7 expressions on pure lineage-negative e-MDSCs. The limited numbers of patients and healthy donors included in this study preclude meaningful statements about possible differences between Siglec expression on MDSCs from patient(group)s or healthy donors.

In addition to expressing Siglec receptors, both MDSC subsets also expressed α2,3-linked and α2,6-linked sialic acids. M-MDSC showed overall higher expression levels of sialic acids compared with PMN-MDSCs. So far, the role of sialic acids in MDSC biology is largely unknown. Our findings suggest that these sialic acids could serve as *cis* or *trans* ligands for Siglec-5, -7 and -9 receptors on the surface of MDCS or other immune cell subsets interacting with MDSCs. Staining of MDSCs with recombinant Siglec-2, -3 and -10 Fc chimeras was negative, yet putative sialic acid ligands for these Siglecs could be masked [43]. Future studies should investigate the role of *cis* (MDSCs) and *trans* (tumor) sialic acid–Siglec interactions in MDSC biology and their relevance for cancer immunotherapy.

Most Siglecs have one or more ITIM motifs allowing them to modulate immune cell activation and function. In MDSCs, Siglec-3 signaling has been shown to induce secretion of suppressive cytokines after forming a functional ligand–receptor pair, for example, with S100A9 [19]. Whether this interaction is sialic acid dependent has not been investigated [19]. We have previously shown that serum from glioma patients contains increased levels of S100A8/9 compared to healthy individuals, suggesting that MDSCs in glioma patients could gain a more suppressive phenotype due to enhanced Siglec-3–S100A9 interactions [13]. On patient-derived glioma cells, we could only detect very low levels of ligands for Siglec-3; so, this is not very likely to affect MDSC function. In addition, we cannot detect Siglec-3 on intra-tumoral PMN-MDSCs, which could be caused by Siglec-3 downregulation or be a consequence of the isolation process. However, we did observe a clear expression of Siglec-7 and -9 ligands on glioma cells ex vivo as well as in situ and the corresponding Siglecs are present on both blood MDSCs and (at least Siglec-9) on tumor-infiltrated PMN-MDSCs. These findings indicate that glioma cells could, thus, interact with MDSCs via the sialic acid–Siglec-9 axis. It has been shown for other immune cell types that sialic acid–Siglec-7/-9 interactions can dampen immune cell activation or effector function thereby shaping the immunosuppressive tumor microenvironment [25, 29, 31–33, 35, 37, 44]. Further research needs to determine whether glioma sialic acids can indeed interact with Siglec-7 and -9 on MSDCs and/or other myeloid cells in the tumor microenvironment and what the functional consequences are.

In conclusion, our data show that MDSCs from glioma patients and healthy donors express Siglec-3, -5, -7 and -9. On patient-derived glioma cells, ligands are found for Siglec-7 and -9, supporting possible sialic acid-dependent interactions between glioma cells and MDSCs in the tumor microenvironment. It may be highly rewarding to further elucidate whether this interaction occurs in the tumor microenvironment and whether sialic acid–Siglec interactions influence the suppressive function of MDSCs. Such studies may provide new opportunities to interfere with MDSC function and may potentiate anti-glioma immunotherapy.

### Electronic supplementary material

Below is the link to the electronic supplementary material.

Supplementary material 1 (PDF 130 kb)

## Abbreviations

e-MDSC: Early-stage myeloid-derived suppressor cell
IDH: Isocitrate dehydrogenase
ITIM: Immunoreceptor tyrosine-based inhibitory motif
M-MDSC: Monocytic myeloid-derived suppressor cell
MALII: *Maackia amurensis* lectin II
PMN-MDSC: Polymorphonuclear myeloid-derived suppressor cell
PNA: Peanut agglutinin
Radboudumc: Radboud University Medical Center
Siglec: Sialic acid-binding immunoglobulin-like lectin
SNA-I: *Sambucus nigra* agglutinin
S100A8: S100 calcium-binding protein A8
