# Supplementary material for: Expression profiling of immune inhibitory Siglecs and their ligands in patients with glioma
Source: Cancer Immunol Immunother. 2019 Apr 5;68(6):937–49. doi: 10.1007/s00262-019-02332-w (PMC6529385; doi:10.1007/s00262-019-02332-w)
Supplement: Supplementary file 1 — Supplementary material 1 (PDF 130 kb) [file 262_2019_2332_MOESM1_ESM.pdf]

SIGLEC	FW Primer	RV Primer
SIGLEC1	CCTCGGGGAGGAACATCCTT	AGGCGTACCCCATCCTTGA
SIGLEC2	GCACCCTGAAACCCTCTACG	ATCAAACTTCGAGGTGTTCTTGT
SIGLEC3	GCCCCAGGACTACTCACTC	CCAGCGAACTTCACCTGACA
SIGLEC5	TTCAGGAACGGCATAGCCCTA	TACTCGACGAAGCTCCAAGAT
SIGLEC6	AGCCTCGTACTATGGTTATGGC	CACTTCTTCGTCTGGGTCGTT
SIGLEC7	CGGAAGGATTACTCGCTGACG	CCAGCTTATATCATTCCCTGCC
SIGLEC8	CAATATGGGGATGGTTACTTGCT	GGAGCGTCTTGGTATGGTCTG
SIGLEC9	CCACATACCAAGAATTGCACCC	ACAGAGAGCCGGTGATGTTTAT
SIGLEC10	AAGGGACTCATCTCAACGGC	CCGTCTCTTCGGTAGAATCTTCA
SIGLEC11	CTACTGCTGCTTATGGCTACTG	CGAAAGAAGTACCATGCCTCATC
SIGLEC14	CACTGACTACCCCTCTTTTC	CGAAAGTACATTCCCTTCAC
SIGLEC15	CGCGGATCGTCAACATCTC	GTTCGGCGGTCACTAGGTG
SIGLEC16	GGCTAATCAGAAACTCCAAA	AGGGAGACTTGAGACATGAG

Supplementary Table 1 qPCR primers for the human Siglec family

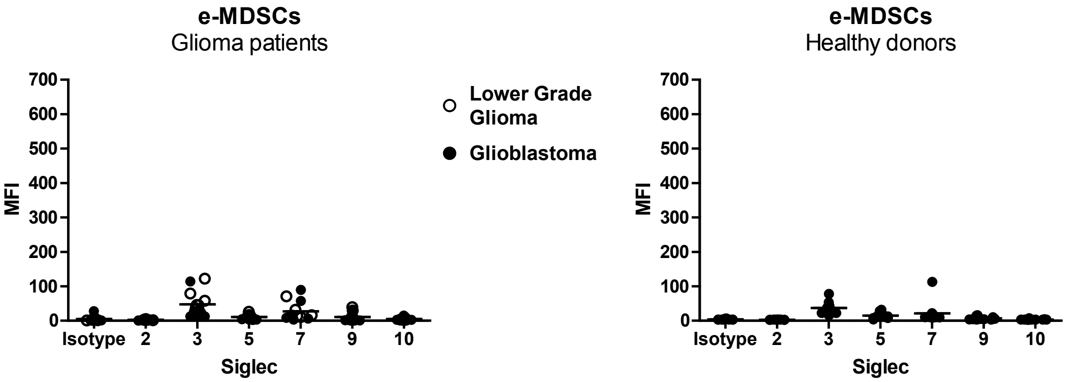

Supplementary Fig. 1 Siglec expression on e-MDSCs from glioma patients and healthy donors. PBMCs freshly isolated from blood of glioma patients (n=13; 6 lower grade gliomas (open circles) and 7 glioblastomas (filled circles)) and healthy donors (n=14) were stained as described and $CD33^{+}$ MHC $II^{-}CD15^{-}CD14^{-}$ cells were gated as shown in **Figure 1a**. E-MDSCs were analyzed for Siglec expression. Horizontal lines show the mean Siglec expression.

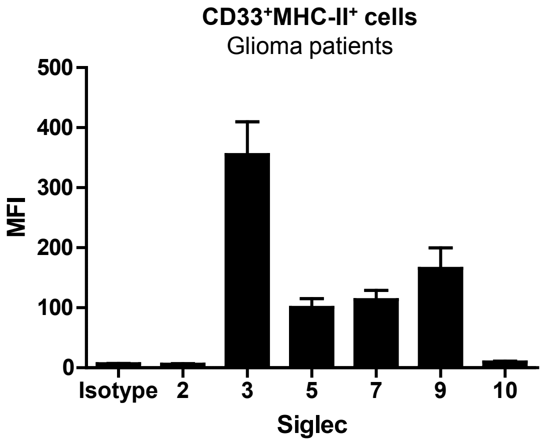

Supplementary Fig. 2 Siglec expression on $CD33^+$ MHC II^+ PBMCs from glioma patients. PBMCs freshly isolated from blood of glioma patients (n=9) were stained as described and single, viable cells were gated based on CD33 and MHC II expression as shown in **Figure 1a**. $CD33^+$ MHC II^+ cells were further analyzed for Siglec expression. Bar diagram shows pooled data of cell surface Siglec expression as mean fluorescence intensity ± SEM.
